# Wheelchair services and use outcomes: A cross-sectional survey in Kenya and the Philippines

**DOI:** 10.4102/ajod.v6i0.318

**Published:** 2017-10-20

**Authors:** Eva S. Bazant, Elizabeth J. Himelfarb Hurwitz, Brenda N. Onguti, Emma K. Williams, Jamie H. Noon, Cheryl A. Xavier, Ferdiliza D.S. Garcia, Anthony Gichangi, Mohammed Gabbow, Peter Musakhi, R. Lee Kirby

**Affiliations:** 1 Jhpiego, Baltimore, United States; 2 Jhpiego, Nairobi, Kenya; 3 Noon Design, Cerrillos, United States; 4 Private Sector, Matale, Sri Lanka; 5 College of Allied Medical Professions, University of the Philippines, Manila, Philippines; 6 National Council for Persons With Disabilities, Government of Kenya, Kenya; 7 Ministry of East African Community (EAC), Labour and Social Protection, Government of Kenya, Kenya; 8 Division of Physical Medicine & Rehabilitation, Dalhousie University, Canada

## Abstract

**Background:**

The World Health Organisation recommends that services accompany wheelchair distribution. This study examined the relationship of wheelchair service provision in Kenya and the Philippines and wheelchair-use–related outcomes.

**Method:**

We surveyed 852 adult basic manual wheelchair users. Participants who had received services and those who had not were sought in equal numbers from wheelchair-distribution entities. Outcomes assessed were daily wheelchair use, falls, unassisted outdoor use and performance of activities of daily living (ADL). Descriptive, bivariate and multivariable regression model results are presented.

**Results:**

Conditions that led to the need for a basic wheelchair were mainly spinal cord injury, polio/post-polio, and congenital conditions. Most Kenyans reported high daily wheelchair use (60%) and ADL performance (80%), while these practices were less frequent in the Philippine sample (42% and 74%, respectively). Having the wheelchair fit assessed while the user propelled the wheelchair was associated with greater odds of high ADL performance in Kenya (odds ratio [OR] 2.8, 95% confidence interval [CI] 1.6, 5.1) and the Philippines (OR 2.8, 95% CI 1.8, 4.5). Wheelchair-related training was associated with high ADL performance in Kenya (OR 3.2, 95% CI 1.3, 8.4). In the Philippines, training was associated with greater odds of high versus no daily wheelchair use but also odds of serious versus no falls (OR 2.5, 95% CI 1.4, 4.5).

**Conclusion:**

Select services that were associated with some better wheelchair use outcomes and should be emphasised in service delivery. Service providers should be aware that increased mobility may lead to serious falls.

## Introduction

An estimated 20 million people worldwide in 2003 needed a wheelchair for mobility and lacked one (World Health Organization [WHO] 2008). In less resourced settings, access to appropriate wheelchairs is limited. An appropriate wheelchair allows the user to meet his or her mobility needs in the local environment, providing postural support with proper fit and is durable, safe, available, affordable and maintainable by the user (WHO 2008). Globally, charitable, governmental and service organisations provide wheelchairs. However, users in less resourced settings often receive inappropriate wheelchairs or wheelchairs with inadequate services. Awareness is increasing wheelchair provision by trained personnel increases the chance that wheelchair users receive appropriate wheelchairs (Toro, Eke & Pearlman 2016).

The WHO Guidelines on the Provision of Manual Wheelchairs in Less Resourced Settings calls for the components of wheelchair service delivery to include referral and appointment, assessment, prescription (selection), funding and ordering, product preparation, fitting, user training, follow-up, maintenance and repairs (WHO 2008). Few studies have assessed whether users in less resourced settings have received these services and user outcomes (Borg et al. 2012; Greer, Brasure & Wilt 2012; Toro et al. 2016).

Lack of service provision may be one of the reasons for wheelchair abandonment. In 2005 in West Bengal, India, over half of 162 hand rim–propelled manual wheelchairs distributed to individuals with lower-limb dysfunction went unused because of pain, fatigue, discomfort and lack of habitat adaptability (Mukherjee & Samanta 2005). It is not known if the users who abandoned these wheelchairs had received any supportive services.

Wheelchairs need to be rugged to withstand use in challenging terrain (Rispin &Wee 2014). In Nepal, two-thirds of donated standard wheelchairs in one study needed replacement within two years, leaving users unable to access the community independently (Scovil et al. 2012). Even in the United States, about half of wheelchairs need repair within six months of issue (Worobey et al. 2012).

User involvement in wheelchair selection has seen positive effects. In Bangladesh, measuring a user for a wheelchair increased the likelihood of wheelchair satisfaction, and wheelchair training was associated with the user reporting fewer activity limitations and participation restrictions (Borg et al. 2012). The effectiveness of wheelchair skills training is well documented (for instance Best et al. 2005, 2016; Kirby et al. 2016a; MacPhee et al. 2004; Ozturk & Ucsular 2011; Routhier et al. 2012; Worobey et al. 2016; Tu et al. 2017). However, greater wheelchair use in varied terrain can lead to falls and injury (Berg, Hines & Allen 2002; Calder & Kirby 1990; Gaal et al. 1997; Kirby et al. 1994; Nelson et al. 2010; Xiang, Chany & Smith 2006).

The objective of this study was to examine whether wheelchair service receipt is associated with successful wheelchair use among adult, basic manual wheelchair users in less resourced settings. The hypothesis was that wheelchair service receipt is associated with high daily wheelchair use, independent outdoor mobility and high performance of activities of daily living (ADL) and with fewer reports of serious falls.

## Research methods and design

### Design and locations

This was a cross-sectional survey of wheelchair users in Kenya and the Philippines. The two-country study was done in sub-Saharan Africa and Asia at the request of the funder. Wheelchair sector stakeholders advised the research team on the study location. Priority was placed on countries with high wheelchair distribution, countries with organisations providing wheelchair services and a country where the institution of the lead author of this article had an office. In each country, investigators met with stakeholders in the field of disability and wheelchairs to discuss the study purpose and methods.

### Dissemination of information about the study to organisations, sampling and recruitment

We aimed to enrol a sample that would be composed equally of participants who had received services with the current wheelchair and those who did not.

Eligible wheelchair users were age 18 or older, did not require postural support and those who received their current or most recently acquired wheelchair within the past five years but greater than three months before the survey date to ensure experience with the current chair. Exclusion criteria were being a temporary wheelchair user or a user of an arm-crank–propelled tricycle, inability to communicate in either English, Swahili in Kenya or Filipino in the Philippines; or inability to understand the questions. In the Philippines, it was difficult to find users who had received services. In the last month of data collection, the study protocol was amended to include wheelchair users who had received the current wheelchair in the last 10 years.

The screening question to potential participants was: ‘When you received your current or most recent chair, did a wheelchair provider help you choose the right wheelchair? The provider might have measured your body, checked the fit of the wheelchair, or made adjustments to the wheelchair.’

In Kenya, residents of urban and peri-urban areas were sampled from lists provided by wheelchair-providing organisations that were faith-based, non-governmental, community-based, related to disabled person’s rights or government hospitals and schools. In the Philippines, Greater Manila residents were sampled through lists of wheelchair users provided by five local government units that provided wheelchairs to citizens, a wheelchair charity and a non-governmental organisation that employed wheelchair users. In addition, in both countries, snowball sampling occurred, in which study participants referred members of their personal networks. Participants and up to one caregiver received locally appropriate participation and travel reimbursements in the Philippines. In Kenya, local investigators considered reimbursement inappropriate and it was not given in Kenya, per the study protocol and this was approved by the ethical review boards.

### Sample size

The sample size calculation was specified to detect a difference of 15% points on the primary outcome of high daily wheelchair use between wheelchair users who reported receiving services with the current chair and those who did not. The hypothesis was that the outcome would be 50% among those in the service group, a percentage that would yield the largest and most conservative sample size. The power was set at 80%, alpha level of significance was set at 0.05 and intra-cluster correlation was set at 0.002. The sample size calculation called for 500 per country with 10 clusters or groupings.

### Data collection and management

Data collection occurred from December 2014 to June 2015 in Kenya and from February to May 2015 in the Philippines. In Kenya, the authors’ institutional staff collected data. In the Philippines, a local organisation with experience in disability sector research was selected through a competitive process. Data collection was conducted by 8 surveyors and 4 field supervisors in Kenya and 15 surveyors and 7 field supervisors in the Philippines. Surveyors had secondary education and data collection experience in the Philippines and college/university education in Kenya. In each country, surveyors received one week of training on data collection procedures, including use of the instruments, informed consent and research ethics.

The paper-based tool was digitised using Open Data Kit (https://opendatakit.org/ and Brunette et al. 2013) and collected on Android tablet computers in each country. Data were exported to a Microsoft Excel 2013 database. Data cleaning was conducted in Kenya using Excel and in the Philippines in IBM Statistical Product and Service Solutions version 20 for Windows (Armonk, NY, 2011). This step was to ensure that study identification numbers for the participants were captured correctly and were not repeated and that snowballed participants were assigned to the index participant’s cluster for analysis.

### Instrument development

The development of the survey instrument occurred in phases. Firstly, the investigators sought out published instruments and 22 instruments were found. Secondly, the content of the questions in the instruments was reviewed for the main themes and sub-themes. Many of the existing instruments were not considered to be readily adaptable to the setting and research questions of this study within the time frame of the project. Some questions used in the survey were informed by the existing instruments. These instruments include the Wheelchair Adapted International Outcome Inventory for Hearing Aids (Borg et al. 2012); the Wheelchair Skills Test Questionnaire (Kirby et al. 2016b); the Wheelchair Use Confidence Scale for Manual Wheelchair Users (Version 3.0) (Rushton et al. 2013); the Efficiency of Assistive Technology and Services 6D Forms (Andrich et al. 1998); and Life Space Assessment (Peel et al. 2005). Throughout the process, wheelchair stakeholders advised on the survey instrument development.

**FIGURE 1-A1:**
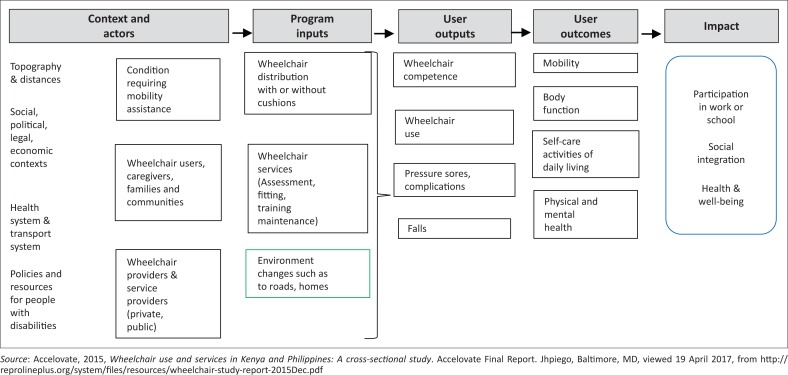
Wheelchair services conceptual framework, Accelovate project.

Thirdly, this study developed a conceptual framework to guide measurement (Figure 1-A1; Accelovate 2015). It was informed by two main published frameworks, a framework of public health intervention researchers (Bryce et al. 2011) and the wheeled mobility framework of Routhier et al. (2003). The study framework covered the environmental context and actors and programme inputs, outputs, outcomes and impact.

**FIGURE 2-A1:**
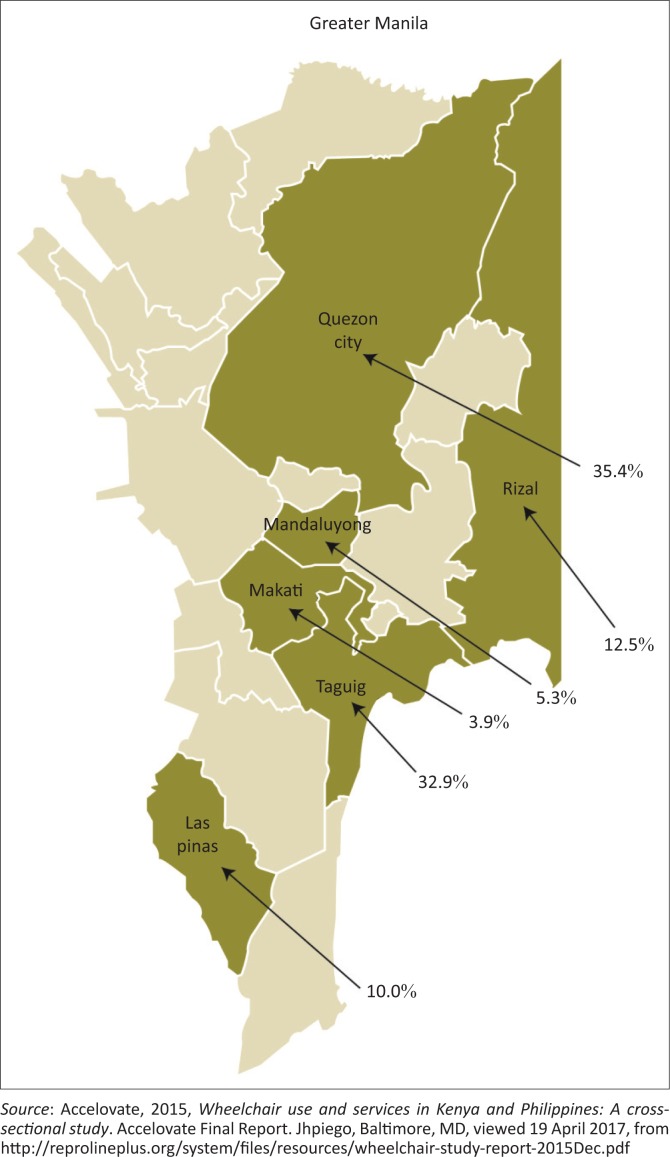
Distribution of the samples by country and geography.

Fourthly, pilot testing of the instrument occurred during field visits in April 2014 in Manila, Philippines, and May 2014 in Dar es Salaam and Moshi, Tanzania, possible locations for the study (Figure 2-A1; Accelovate 2015). Investigators met with organisations providing wheelchairs and services. The draft survey instrument was discussed with wheelchair organisation stakeholders and through a focus group with wheelchair users to discuss the instrument and gather feedback on the relevance of questions to their lives. After the field visits, the survey instrument questions were refined.

The survey instrument asked about past receipt of the steps of wheelchair service recommended by WHO considered most amenable to self-reporting: assessment, fitting, training and maintenance, repair and follow-up (WHO 2008). The survey’s questions were related to sociodemographic and other personal characteristics, the experience and receipt of wheelchair services with the current wheelchair or any wheelchair ever received during the lifetime. The survey questions were also related to the wheelchair characteristics and acquisition. Lastly, the questions inquired about the outputs of daily wheelchair use and experience of severe falls and the outcomes of high performance of ADL and unassisted outdoor mobility. The survey instrument was translated into Swahili in Kenya and Filipino in the Philippines and back-translated to English.

### Variables and analysis

We collected sociodemographic, clinical and wheelchair data, specifically age, gender, county (in Kenya), educational level, marital status, employment type, wealth quintile, condition that led the user to need a wheelchair, number of wheelchairs acquired in last five years, source of wheelchair and whether or not the wheelchair was donated or received at no cost to the user. The question on the type of wheelchair had response categories of ‘basic indoor chair’, ‘rough terrain wheelchair with long wheel-base’, ‘wheelchair unavailable’ and ‘don’t know’. Household wealth was based on a module of standard questions from large household surveys (Kenya National Bureau of Statistics and ICF Macro 2010).

The survey asked whether the user received one of many services with the current wheelchair, as well as services ever received during the participant’s lifetime. In the analysis, the answer of ‘yes’ to several questions was used to create a composite or summary variable to describe assessment, fitting and training. Maintenance, repair, follow-up and other services were assessed in a single item. Details on creation of the service variables are presented in Table 1-A1.

To measure the output of daily wheelchair use, participants were asked how long they used or occupied their wheelchairs from the time they woke up to midday and from midday to the time of going to bed, to arrive at the total number of hours. Responses were categorised as ‘not daily’, ‘1–7 hours daily’ and ‘≥ 8 hours daily’. This was based on the distribution of responses to the continuous variable.

To measure the output of falls, the survey asked, ‘With your current wheelchair have you ever fallen?’. The next question was ‘Was this a serious fall? By serious, I mean a fall that left you with pain or soreness that lasted more than one hour, bruising, skin cuts or abrasions, or injuries to your bones or joints’. This variable was analysed as a three-level variable of ‘no falls’, ‘non-serious falls’ and ‘serious falls’.

To measure the outcome of unassisted outdoor mobility, three survey questions were used. Users were coded as ‘yes’ on this outcome if they reached an area outside their home in a wheelchair in the last month and did not need help in doing so. Those who did not have another area to go to were excluded. Those who reached another area but not in a wheelchair were coded ‘no’.

To measure the outcome of high performance of ADL, participants were told, ‘For each activity that I read, please let me know if you perform it independently or assisted’. Items were bathing, dressing, eating and toilet hygiene. Response categories were ‘Independently’ and ‘Assisted’. If the activity was performed independently or unassisted, this variable was coded 1 and if not, 0. Performance was considered ‘high’ if at least three of the four items were carried out unassisted and ‘low’ for zero-two items.

Analysis was carried out for each country separately and data were not combined across countries because of differences in sampling strategies and geographic coverage. Descriptive results are frequencies and tabulations. In bivariate analysis, each wheelchair service variable was assessed for its association with the output or outcome. Logistic regression was used for dichotomous variables yielding odds ratios (ORs). Multinomial logistic regression was used for three-level outcomes. These models produce relative risk ratios, which can be interpreted as ORs. The models accounted for within-cluster correlation of outcomes (Rogers 1993) using the ‘vce(cluster)’ option in Stata software (StataCorp 2013). In Kenya, the organisation from which the investigators received the contact information of the wheelchair users was designated the cluster. In Philippines, a neighbourhood unit called a *barangay* within the local government unit from which we received the contact information of the wheelchair users was designated the cluster. In both countries, ‘snowballed’ wheelchair users who were referred by another participant to the survey team were assigned the cluster of the index participant.

To answer the research questions, wheelchair service items as well as participants’ sociodemographic or wheelchair user variables significantly associated with wheelchair use outputs and outcomes at *p* < 0.10 in the bivariate analyses were entered into multivariable models. Multivariable regression models controlled for potential confounders of the relationship of wheelchair services and wheelchair outputs or outcomes.

Models in Kenya were adjusted for number of wheelchairs acquired in the last five years, county, age category, educational level, marital status, employment type, condition that led the user to need a wheelchair, source of wheelchair, wealth quintile, whether or not the wheelchair was donated and type of wheelchair. Models in Philippines were adjusted for number of wheelchairs acquired in last five years, region, age category, educational level, gender, condition that led user to need a wheelchair, any employment, source of wheelchair, and whether or not the wheelchair was donated. Models for performance for ADL and falls were also adjusted for the three-level daily wheelchair use level.

A few variables had some missing data. In Kenya, between 10 and 12 respondents were missing data for age and marital status. In the Philippines, the source of the wheelchair was missing for 10 respondents and for ‘current wheelchair donated’, 13 are missing. For ‘current wheelchair has a cushion’, the interviewer was unable to record this information for 60 respondents in Kenya and 5 respondents in the Philippines. Wheelchair services significantly associated with each outcome were identified by the 95% confidence interval (CI) of the adjusted ORs that did not cross 1.0. All analyses were conducted in Stata 13.0 (StataCorp, College Station, TX).

### Ethical consideration

This study received ethical approval from the institutional review boards of Johns Hopkins University Bloomberg School of Public Health in Baltimore, MD, United States (#5839); Kenya Medical Research Institute in Nairobi, Kenya (Non-SSC Determination #457); and University of Philippines Manila Research Ethics Board (#2014-351-01). All study participants provided informed consent immediately prior to the survey participation. Consent was oral in Kenya, according to an approved consent script. At the request of the University of Philippines, consent included participant signature. Interviews were carried out in spaces allowing for audio and visual privacy.

## Results

### Samples achieved

In Kenya, after removing duplicate names from a list of 1764 wheelchair users, potentially eligible participants were 1612; of these participants, 671 could not be reached. Of the 941 participants screened, 512 were eligible and 429 ineligible. Of the eligible, 72 participants were unavailable. In Kenya, 440 participants were included. Twenty participants were younger than age 18 according to the birthdate in the survey and their data were not analysed, yielding 420 participants whose data were analysed.

**FIGURE 3-A1:**
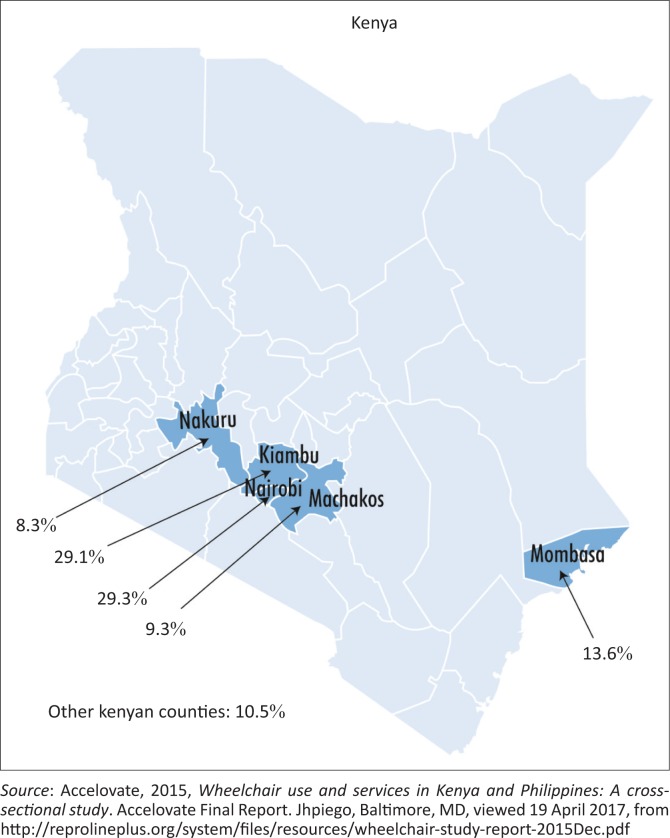
Distribution of the samples by country and geography.

In the Philippines, of the 1490 wheelchair user names, 575 potential participants could not be screened because they were inaccessible by phone or because they had died. Of the 915 participants reached and screened, 497 were eligible and 417 ineligible because they used a non-basic wheelchair, they were younger than age 18, were unable to communicate or had received current wheelchair more than five years ago. However, this criterion was relaxed in the last month of survey data collection to reach enrolment goals. In the Philippines, 56 participants were unavailable and 9 declined. Overall, 432 participants were included. Figure 3-A1 depicts the locations of the study participants in each country.

### Descriptive results

#### Sample characteristics

In Kenya, participants were primarily men, younger than age 50 and employed (Table 1). The most commonly reported conditions requiring the need for wheelchairs were spinal cord injury, polio or post-polio and congenital issues. The most common sources of the current wheelchairs were charity, government and family or friend. Current wheelchair types were basic indoor wheelchairs for the majority of the sample, while others had rough-terrain wheelchairs or unknown types. For 54% of participants, the current wheelchairs had a cushion at the time of the interview.

**TABLE 1:** Sample characteristics in Kenya and the Philippines.

Characteristics	Kenya (*n* = 420) %	Philippines (*n* = 432) %
**Gender**		
Male	59.8	50.2
Female	40.2	49.8
Age		
18–34	39.2	12.5
35–49	32.4	24.6
50+	28.4	62.9
**Education**		
None	7.6	3.0
Primary	31.2	32.4
Secondary, Post-secondary, Vocational	38.6	36.8
College or University	22.6	27.8
**Marital status**		
Married or Cohabiting	42.2	49.5
Never married/Never cohabiting	49.0	25.2
Divorced, Separated or Widowed	8.8	25.2
**Employment**		
No work or Unemployed	28.1	61.3
Trading or Selling	18.6	7.9
Student	14.5	2.3
Craftsman	12.9	6.9
Office worker	7.9	4.2
Other	18.1	17.4
Any work (% yes)	71.9	39.1
**Condition related to need for wheelchair**		
Spinal cord injury: Paraplegia or Quadriplegia	28.8	10.0
Polio or Post-polio	23.8	19.2
Congenital	13.1	7.2
Old age, Arthritis, Bone problems	0	14.8
Stroke, or Nerve, or Clot mentioned	0	26.4
Other	34.3	22.5
**Source of current wheelchair**		
Government	17.1	48.3
Mission hospital	9.1	2.6
Charity	37.6	21.1
Pharmacy or Store	3.8	9.0
Friend or Family	16.9	13.7
Other	15.5	5.2
Current wheelchair was donated/ or received at no cost (% yes)	79.9	77.6
**Current wheelchair type**		
Basic indoor wheelchair	58.1	91.4
Rough terrain wheelchair	27.1	3.9
Wheelchair unavailable or Don’t know	14.8	0.7
Other	0.0	3.9
Current wheelchair has a cushion (% yes)	63.6	27.6

*Source*: Authors’ own work

*n*, number.

In the Philippines, participants were about equally split by gender, mostly age 50 or older and unemployed, and nearly half were married or cohabiting. Conditions related to the need for wheelchairs included old age, arthritis and bone problems; polio or post-polio and spinal cord injury plus old age or arthritis or bone problems, and stroke, nerve- or clot-related problems. Most received their wheelchairs from government, charity or family or friend. For most users, the current wheelchairs were basic indoor wheelchairs (91%) and few wheelchairs had cushions (28%). In both countries, most users received the wheelchair at no cost. Because of a concerted effort to find users who received services and the expansion of eligibility criterion, 16% of the sample in the Philippines had received their chair more than 5 and less than 10 years before the survey.

#### Wheelchair services received

In each country, approximately 40% of the participants were classified in the service-received category according to the response to the screening question. In Kenya, for the current wheelchair, a third of participants received wheelchair assessment (31%), a third of participants received wheelchair fitting (34%) and 42% were fitted while the user propelled the wheelchair (Table 2). Few participants received any other services with the current wheelchair. Regarding services ever received in the lifetime, just over a quarter of participants received wheelchair training; similarly, 26% ever received instructions in taking care of the wheelchair; 41% reported that a provider had ever helped choose the right wheelchair. Few participants had ever been told where to seek help with repairs (15%) or had ever been contacted by the provider in follow-up (15%). The question on skin problems is not reported on because of an issue with the programming of this item in the software.

**TABLE 2:** Wheelchair services received in Kenya and the Philippines.

Variable	Kenya (*n* = 420) %	Philippines (*n* = 432) %
Services received with the current wheelchair		
The wheelchair provider…		
…did assessment on 2+ aspects (vs 0–1)	30.5	31.0
…did fitting (any of 5 items)	33.6	26.4
…fitted the wheelchair while user propelled wheelchair	41.9	39.1
…asked or physically checked user for skin problems or pressure sores	23.6	14.8
…checked for unsafe pressure at seat surface	14.1	10.4
…did the assessment or fitting at the user’s home	6.9	22.9
…took 30+ min to assess†	21.4	13.5
**Services ever received**		
The wheelchair provider…		
…did training (any of 4 items referring to training)	26.7	17.1
…ever helped user choose the right wheelchair	41.0	39.6
…ever instructed user in taking care of the wheelchair, such as keeping it clean, oiling moving parts, tightening spokes or pumping tires	25.5	26.4
…ever told user where to seek help with wheelchair repairs	14.5	17.6
…ever contacted user to ask how she or he was doing with the wheelchair	14.8	19.7
Peer group training ever received	14.3	12.7

*Source*: Authors’ own work

† , In Kenya, four respondents were missing.

*n*, number.

In the Philippines, for the current wheelchair nearly a third of participants received wheelchair assessment (31%), a quarter received wheelchair fitting (26%), and 39% were fitted while the user propelled the wheelchair. Few participants (15%) reported that a provider had asked about or physically checked the user for skin problems or pressure sores; 10% were checked for unsafe seat pressure. Regarding services ever received in the lifetime, 17% of participants had received wheelchair-related training, a quarter of participants received provider instructions in taking care of the wheelchair and 40% reported that a provider had ever helped choose the right wheelchair. Few participants had been told where to seek help with repairs (18%) or were contacted by the provider (20%).

#### Wheelchair-related outputs and outcomes

In Kenya, most participants (60%) reported using the wheelchair daily for 8 h or more and 80% independently performed at least three of the four assessed ADL (Table 3). Only 25% of participants used their wheelchairs outdoors unassisted in the past month. Falls were common; 22% had ever had a serious fall, while 37% reported a non-serious fall.

**TABLE 3:** Wheelchair use outputs and outcomes in Kenya and the Philippines.

Output/Outcome	Kenya (*n* = 420) %	Philippines (*n* = 432) %
**Daily wheelchair use**		
Not daily	17.0	41.7
1–7 h	23.4	16.2
8+ h	59.7	42.1
**Fall(s) in current wheelchair**		
None	41.5	66.0
At least one non-serious fall	37.1	21.6
At least one serious fall	21.5	12.4
High performance of activities of daily living, unassisted	80.0	73.3
Outdoor unassisted wheelchair use	25.4	33.3

*Source*: Authors’ own work

*n*, number.

In the Philippines, 42% of participants used their wheelchairs for 8 h or more and 16% used it 1–7 h daily. Most participants (73%) independently performed at least three of four ADL. A third (33%) of participants used their wheelchairs outdoors unassisted. Two-thirds of Filipino users had not fallen (66%).

### Bivariate results

Daily wheelchair use was associated at *p* < 0.05 with one service variable in Kenya, ‘provider ever instructed user in taking care of wheelchair’. In the Philippines, daily wheelchair use was associated at *p* < 0.05 with 10 service variables: ‘assessment with current chair’, ‘fitting’, ‘assessment of wheelchair fit while user propelled wheelchair’, ‘provider asking or physically checking user for skin problems, sensation or pressure sores’, ‘assessment duration: < 30 minutes’, ‘training ever received’, ‘provider ever helped user choose the right wheelchair’, ‘provider ever instructed user in taking care of wheelchair’, ‘provider ever told user where to seek help with repairs’ and ‘peer group training’.

In Kenya, reporting at least a non-serious fall versus no falls at the bivariate level was associated with three service variables: ‘assessment duration of less than 30 minutes’, ‘provider ever helped user choose the right wheelchair’ and ‘provider ever instructed user in taking care of wheelchair’. In the Philippines, reporting a non-serious fall versus no falls was associated with the same 10 service variables as for wheelchair use.

High performance of ADL was associated at *p* < 0.05 with three service variables in Kenya: ‘provider assessed wheelchair fit while user propelled wheelchair’, ‘provider asked or physically checked user for skin problems, sensation or pressure sores’ and ‘training ever received’. High performance of ADL was associated with nine service variables in the Philippines. These were the same as for daily wheelchair use, except for ‘provider ever helped user choose the right wheelchair’.

Outdoor unassisted wheelchair use was associated with three service variables in Kenya: ‘assessment’, ‘assessment of wheelchair fit while user propelled wheelchair’ and ‘training ever received’. In the Philippines, outdoor unassisted wheelchair use was associated with the same 10 service variables as for wheelchair use.

### Multivariable model results

The wheelchair use outputs associated with wheelchair service items in multivariable models are presented in Table 4.

**TABLE 4:** Wheelchair use outputs (daily wheelchair use and falls) and services received in Kenya and the Philippines, adjusted odds ratios (aOR) and 95% confidence intervals from multivariable regression.

Services received	Daily wheelchair use				Falls			
	Low versus No	High versus No	Low versus No	High versus No	Non-serious versus None	Serious versus None	Non-serious versus None	Serious versus None
	Kenya (*n* = 392)		Philippines (*n* = 405)		Kenya (*n* = 387)		Philippines (*n* = 416)	
**Services with the current wheelchair**								
Assessment on 2+ aspects	†	†	0.8 (0.3, 2.4)	1.2 (0.6, 2.4)	1.2 (0.5, 2.7)	1.1 (0.3, 3.4)	0.8 (0.3, 2.3)	0.5 (0.2, 1.3)
Assessment took 30+ min versus 0–29 min	†	†	0.7 (0.1, 5.6)	0.5 (0.3, 1.0)	1.6 (0.8, 3.1)	1.7 (0.6, 4.4)	0.9 (0.3, 2.8)	2.4 (1.3, 4.5)*
Provider asked or checked user for skin problems	†	†	2.1(0.7, 6.4)	0.5 (0.2, 0.97)*	†	†	0.3 (0.1, 1.3)	1.6 (0.4, 6.7)
Fitting of wheelchair (any)	1.2 (0.4, 3.7)	0.9 (0.3, 2.4)	1.2 (0.5, 3.3)	1.3 (0.6, 2.6)	†	†	0.8 (0.5, 1.5)	0.7 (1.4, 4.5)
Assessment or fitting occurred at home	†	†	†	†	†	†	†	†
Provider checked for unsafe pressure at seat	†	†	†	†	†	†	†	†
Fit assessed while user propelled wheelchair	†	†	1.2 (0.5, 3.2)	1.0 (0.6, 1.7)	†	†	0.6 (0.3, 1.5)	0.2 (0.0, 1.3)
**Services ever received**								
Provider ever helped user choose wheelchair	†	†	2.1 (0.6, 7.4)	2.8 (1.1, 6.9)*	0.9 (0.6, 1.4)	0.7 (0.3, 1.7)	2.2 (0.6, 8.2)	2.4 (0.6, 9.4)
Training in wheelchair (any, ever)	0.8 (0.2, 3.3)	0.9 (0.2, 4.0)	0.8 (0.2, 3.1)	4.0 (2.3, 7.0)*	†	†	1.7 (0.8, 3.5)	2.5 (1.4, 4.5)*
Peer group training ever received	†	†	1.5 (0.2, 10.6)	2.1 (0.7, 6.4)	†	†	2.3 (0.8, 6.3)	2.1 (1.1, 4.0)*
Ever instructed in caring for wheelchair	2.7 (0.9, 7.8)	3.3 (1.0, 10.5)	0.4 (0.2, 0.7)*	0.5 (0.2, 0.9)*	1.6 (0.8, 3.1)	1.5 (0.7, 3.2)	1.1 (0.5, 2.5)	0.6 (0.2, 1.7)
User ever told where to seek repairs	†	†	0.6 (0.1, 5.0)	1.0 (0.3, 3.2)	†	†	3.5 (1.0, 12.1)*	6.1 (2.0, 18.4)*
Provider ever contacted user regarding wheelchair	0.8 (0.4, 1.9)	0.4 (0.2, 0.9)*	†	†	†	†	0.4 (0.2, 1.0)	1.7 (0.8, 3.6)

*Source*: Authors’ own work

The cells show adjusted odds ratios (aOR) and 95% confidence intervals from multinomial logistic regression models accounting for clustering. The odds ratio is significant if the confidence interval does not cross 1.0.

* , findings indicate statistical significance.

† , indicates that the service variable was insignificant at the bivariate level (*p* > 0.05) and was not entered to multivariable model.

In Kenya, one service item was associated with daily wheelchair use: the provider ever contacting the user about the wheelchair use was associated with *reduced* odds of daily wheelchair use (OR 0.4, 95% CI 0.2, 0.9). No service item was associated with odds of falls.

In the Philippines, four service items were associated with daily wheelchair use. Ever receiving wheelchair training was associated with 4-fold increased odds of high versus no daily use (95% CI 2.3, 7.0). A provider helping the user choose the right wheelchair ever was associated with 2.8-fold increased odds of high versus no daily use (95% CI 1.1, 6.9). However the provider of the current wheelchair asking about or checking user for skin problems, sensation or pressure sores was associated with *reduced* odds of high versus no daily use (OR 0.5, 95% CI 0.2, 0.97). Ever being instructed in taking care of wheelchair was also associated with *reduced* odds of high versus no daily use (95% CI 0.2, 0.91).

In the Philippines, four service items were associated with increased odds of falls. Ever being told where to seek repairs was associated with 6.1-fold increased odds of serious versus no falls (95% CI 2.0, 18.4). Longer assessment duration was associated with increased odds of serious versus no falls (95% CI 1.3, 4.5). Ever receiving wheelchair training was associated with 2.5-fold increased odds of serious versus no falls (95% CI 1.4, 4.5). Peer group training was associated with 2.1-fold increased odds of serious versus no falls (95% CI 1.1, 4.0).

Wheelchair service items associated with outcomes of high performance of ADL and unassisted outdoor use in multivariable models are presented in Table 5.

**TABLE 5:** Wheelchair use outcomes (outdoor unassisted use and activities of daily living) and services received in Kenya and the Philippines, adjusted odds ratios (aOR) and 95% confidence intervals from multivariable regression.

Services received	Outdoor unassisted use		Activities of daily living	
	Yes versus No		High versus Low	
	Kenya (*n* = 382)	Philippines (*n* = 403)	Kenya (*n* = 391)	Philippines (*n* = 403)
**Services with the current wheelchair**				
Assessment on 2+ aspects	†	1.0 (0.4, 2.2)	†	1.4 (0.8, 2.6)
Assessment took 30+ min versus 0–29 min	†	1.0 (0.3, 3.2)	†	1.1 (0.2, 5.1)
Provider asked or checked user for skin problems	†	0.9 (0.3, 3.2)	1.7 (0.7, 4.0)	†
Fitting of wheelchair (any)	1.6 (1.0, 2.7)	1.0 (0.5, 2.1)	†	0.5 (0.2, 1.0)
Assessment or fitting occurred at home	†	0.6 (0.4, 0.9)*	†	†
Provider checked for unsafe pressure at seat	†	‡	†	†
Fit assessed while user propelled wheelchair	1.61 (1.0, 2.7)	2.4 (1.5, 4.1)*	2.8 (1.6, 5.1)*	2.8 (1.8, 4.5)*
**Services ever received**				
Provider ever helped user choose wheelchair	†	1.4 (0.6, 3.3)	†	1.2 (0.4, 3.5)
Training in wheelchair (any, ever)	0.9 (0.5, 1.6)	1.1 (0.4, 2.7)	3.2 (1.3, 8.4)*	0.7 (0.3, 1.8)
Peer group training ever received	†	1.2 (0.4, 3.2)	‡	1.2 (0.4, 3.4)
Ever instructed in caring for wheelchair	†	1.1 (0.5, 2.4)	0.5 (0.2, 1.3)	1.0 (0.4, 2.5)
User ever told where to seek repairs	2.8 (1.5, 5.0)*	1.3 (0.7, 2.5)	†	1.9 (0.5, 7.4)
Provider ever contacted user regarding wheelchair	†	†	†	†

*Source*: Authors’ own work

The cells show adjusted odds ratios (aOR) and 95% confidence intervals accounting for clustering. The odds ratio is significant if the confidence interval does not cross 1.0.

* , findings indicate statistical significance.

† , indicates that the service variable was insignificant at the bivariate level (*p* > 0.05) and was not entered to multivariable model.

In Kenya, three service items were associated with the outcomes ever having been told where to seek repairs, was associated with 2.8-fold increased odds of unassisted outdoor use (95% CI 1.5, 5.0). Two service items were associated with odds of high performance of ADL. Ever being trained was associated with 3.2-fold increased odds of high ADL performance (95% 1.3, 8.4). Having the fit of the wheelchair assessed while the user propelled the current wheelchair was associated with 2.8-fold increased odds of high performance of ADL (95% CI 1.6, 5.1).

In the Philippines, two service items were associated with the outcomes. Having the fit of the current wheelchair assessed while propelling was associated with (1) 2.4-fold increased odds of using the wheelchair outdoors unassisted (95% CI 1.5, 4.1) and (2) 2.8-fold increased odds of high ADL (95% CI 1.8, 4.5), a similar finding to Kenya. However, the provider doing the assessment or fitting at the wheelchair user’s home was associated with *reduced* odds of outdoor unassisted use (OR 0.40, 95% CI 0.4, 0.9).

## Discussion

This study is one of the first to present survey findings from less resourced settings examining specific wheelchair services received and the relationship to user outcomes.

Documenting the effectiveness, efficiency and costs of wheelchair service and distribution programmes is an imperative for funders and programme managers to better meet client needs (Harris & Sprigle 2008). As governments consider how to meet the needs of wheelchair users under national insurance schemes, information on outcomes will be important in establishing coverage priorities. As recommended by Harris and Sprigle (2008), this study in Kenya and Philippines assessed unassisted outdoor mobility and independent performance of ADL and falls.

### Findings, explanations and prior research

Large studies with wheelchair users are rare. In this study in Kenya and the Philippines, wheelchair users who received and did not receive services with their current wheelchairs or in their lifetime were recruited and it was difficult to find users who had received wheelchair services. Similarly, Zongjie et al. (2007) found that while 75% of disabled residents in a Beijing sample expressed a need for rehabilitation, only 27% had received any services. Where wheelchair services are more available, users may be more often informed about or referred to rehabilitation services.

The provider assessing the fit of the wheelchair while the user propelled the wheelchair was associated with greater odds of high ADL performance in both countries. Providers fit clients as they propel in order to determine how the wheelchair user performs certain functions in the wheelchair. When the wheelchair is tailored to the functional needs of the user, performance of ADL may be facilitated.

Training in wheelchair use ever was positively associated with high daily wheelchair use in the Philippines and performance of ADL in Kenya, adding to literature on the safety and effectiveness of wheelchair skills training (for instance Best et al. 2005, 2016; Kirby et al. 2016a; MacPhee et al. 2004; Ozturk & Ucsular 2011; Routhier et al. 2012; Worobey et al. 2016; Tu et al. 2017). Higher odds of reporting falls were associated with receipt of training in Philippines. It may be that trained users had greater confidence to use their wheelchair in new places on rougher terrain or for longer distances without adequate protection and, therefore, incurred more falls. However, it is possible that the nature or dose of the training was suboptimal or the competencies achieved were inadequate. Also, users may have been overconfident following training.

Three provider actions related to wheelchair assessment and fit were negatively associated with three outcomes in the Philippines even while controlling for user characteristics, and several factors may explain this finding. Firstly, in the cultural context, elderly people and people with disabilities may not be expected to be independent, the assistance of a family member or carer is expected, and using of wheelchair outdoors may be associated with stigma in the setting (Tanudtanud-Xavier 2013). Secondly, the environmental context and limited public transport may preclude independent wheelchair mobility. Thirdly, the provider’s actions may have been a *response* to the user’s low daily wheelchair use, outdoor wheelchair use or ADL performance. In a household survey conducted in Beijing, China, having received rehabilitation services was associated with a lower functional independence measure score (Zongjie et al. 2007). The authors concluded that Beijing residents in need of rehabilitation lacking functional independence were seeking out services to improve their situation.

In our study, the user being told where to seek repairs was associated with outdoor unassisted wheelchair use in Kenya and falls in Philippines. Outwardly oriented users may have sought out information on wheelchair repairs. In Philippines, advice on how to repair wheelchairs or get spare parts may have followed chair breakdowns and falls (Williams et al. 2016). Possible factors related to this outcome may be fewer accessible environments and more available compatible parts and repair services.

There were low levels of unassisted outdoor wheelchair use. Generally, in Philippines and urban Kenya, many residential communities for people of limited economic means are not wheelchair accessible. Communities with narrow walkways and small houses may have required wheelchair users to seek assistance from others for mobility.

## Limitations

In a cross-sectional survey, the temporal order of events cannot be confirmed and causality cannot be determined. Questions referring to a past time period may be influenced by recall bias. This study’s results are not directly comparable at this time to other studies. The survey instrument was informed by existing instruments and developed for the specific goals of the study, as was done in other studies of assistive technology (Borg et al. 2012).

Although the survey had many modules to answer key research questions and was comprehensive, certain questions in the survey had limited response possibilities. For example, the question on type of wheelchair’s response category of indoor chair does not indicate if the chair was for short-term hospital transport or intended for longer term use. The outcome of daily wheelchair use does not reflect the users’ functioning while in the wheelchair. It is not possible to know whether wheelchair falls are related to improper wheelchair use.

Generalisability of this study to the national populations of adult, basic wheelchair users is limited by the sampling strategy of recruiting in equal numbers users who received wheelchair services and those who did not. Reported levels of wheelchair services received in this study may be different than those of the national population of wheelchair users, and this can be assessed in a national survey.

### Recommendations for practice or programmes

The study findings were disseminated back to local authorities, wheelchair user and service delivery organisation stakeholders in well-attended meetings in both the Philippines and Kenya, and wheelchair stakeholders were advisers to this study from the beginning. This active engagement of local wheelchair stakeholders and users led to an outpouring of ideas on how to improve policy and programmes.

Training in wheelchair use is related to better wheelchair use outcomes and should continue to be emphasised and delivered in an efficient and equitable manner (Tu et al. 2017). Opportunities should be sought to provide basic wheelchairs partly through peer group workshops, and peer group training should be evaluated (Best et al. 2016). For countries that have an established and working community strategy, engaging community health volunteers can be explored as a mechanism for training wheelchair users.

The provider assessing the fit of the wheelchair (e.g. using the objective version of the Wheelchair Skills Test [Kirby et al. 2016b]) while the user propelled the wheelchair should be an emphasised element of the WHO service package. The provision of wheelchairs and services needs to include a plan for wheelchair maintenance (Toro et al. 2016) and access to spare parts. Wheelchairs should be distributed with a user’s manual and a basic toolkit. Local artisans can be trained to help with maintenance and repair of wheelchairs.

The need for follow-up of wheelchair users and an understanding of their home context and possibility for social and economic integration is paramount, and greater emphasis should be placed on service providers advocating for independent home accessibility for wheelchair users (Scovil et al. 2012. Providers can also engage existing community networks to aid in follow-up of wheelchair users.

Prevention of falls during wheelchair use can be promoted through use of durable chairs with the appropriate weight balance (Toro et al. 2016), fall-avoidance training (Kirby et al. 2016b) and advocacy to change the physical environment to be more manageable to independent wheeled mobility. Self-advocacy training should be added as an element of the WHO service package so that empowered wheelchair users can demand better quality services from service providers, and WHO managers training should be taken up by service managers.

Regarding ADLs, the access to safe water and sanitation affects all persons with special considerations for wheelchair users in less resourced settings (Scovil et al. 2012). WHO should add a service element of counselling in which providers’ explore access to safe water and use of sanitation facilities as an element of activities of daily living.

In the policy domain, governments should establish an evidence-based minimum service package to be delivered along with wheelchairs. Wheelchairs produced locally and imports should be regulated to ensure that all wheelchairs meet specifications for less resourced areas. Providers should consider the costs of wheelchair services when budgeting and should explore public–private partnerships to strengthen the delivery of wheelchairs compliant with standards from the International Standards Organisation.

Tax relief should be enacted to make a wider range of wheelchairs more affordable. Governments should enforce current laws compelling public buildings and transport systems to be accessible to people with disabilities.

Because follow-up with wheelchair users has been largely overlooked, several approaches may be considered: enabling wheelchair provider–initiated contact by embracing eHealth and telemedicine and engaging with community health workers. It may be useful to set up a telephone hotline for wheelchair users. In Romania, a toll-free telephone hotline provides information and referrals to callers on appropriate wheelchairs and services (personal communication between Fundatia Motivation România staff and first author, 07 June 2016).

Providers can make efforts to fit every wheelchair user while the user is propelling the wheelchair. When a user is unable to propel, this may suggest difficulties with unassisted mobility and ADL performance in this wheelchair in the long run. Providers can discuss with clients who request or need at-home services and discuss maintenance and repair with clients. The availability of appropriate wheelchairs and spare parts is imperative at the national level.

### Recommendations for future research

It is recommended for wheelchair service organisations or contracted evaluators to collect contact information from all persons in need of wheelchairs, those who receive wheelchairs and services and their support persons and to keep this information secure and confidential. With additional contact information, service organisations can follow-up with wheelchair recipients over years to ascertain outcomes of wheelchair use and satisfaction, health and well-being, and mortality (Scovil et al. 2012). A long-term study of wheelchair users’ service exposure and outcomes may need to be achieved through funding mechanisms that fall outside that of a typical international development grant period (USAID 2014).

Future population-based surveys of persons with disability should use validated functional measures and plan to further validate scales in the local context, as done by Toro et al. (2016) in Indonesia. The Spinal Cord Independence Measure may be a useful instrument (Anderson et al. 2008) for persons with spinal cord injury. The Wheelchair Skills Test Questionnaire (Kirby et al. 2016b) allows for measurement of wheelchair skills capacity, performance and confidence. Use of Craig Handicap Assessment Recording Technique Short Form (CHART-SF) will allow for measurement of outcomes in multiple domains (Whiteneck et al. 1992).

Wheelchair use outcomes and ADL could be measured by additional means, such as user diaries about the use of different wheelchairs for different activities. Medical records or visits by a study nurse to study participants could help validate reports of adverse health outcomes. Wheelchair use could be measured by accelerometers or global positioning system for measurement of distance travelled or outdoor mobility (Sonenblum et al. 2012). Furthermore, observations by trained observers would provide additional information to understand outcomes (Rispin & Wee 2014).

Kenyan wheelchair stakeholders recommended that future research should incorporate questions targeting wheelchair users in national surveys. Kenyan wheelchair stakeholders would like a study to examine the impact of service receipt on children who use wheelchairs and the costs and affordability of chairs. In addition, questions on wheelchair users can be incorporated into existing standard reporting tools used by community health volunteers of the Ministry of Health. Stakeholders in the Philippines recommended creating a central repository data from various studies involving wheelchair users. It was emphasised that wheelchair users be involved in all phases of research. It is important to evaluate service delivery models as done by Toro et al. (2016).

## Conclusion

In a two-country survey of over 800 adult basic wheelchair users, select services that were associated with some better wheelchair use outputs and outcomes should be emphasised in service delivery. Specifically, assessing the fit of the wheelchair while the user propelled the chair and training in wheelchair use are services associated with positive wheelchair use outcomes. Serious falls may be an unintended consequence of increased mobility. Efforts to provide wheelchairs and services need to include plans for wheelchair maintenance and repair and follow-up with wheelchair recipients.

## Appendix 1

**TABLE 1-A1:** Description of variables.

Variable	Categories	Description
**Outcomes**		
Daily wheelchair use	3-level: high, low, and no	‘How often do you use or occupy your wheelchair?’ (q325) The next question had a preamble: ‘I’d like to ask you some questions about how many hours per day you use or occupy your wheelchair.’ (q326) The first question was: ‘In the morning from the time you wake until midday, how many hours are you in the wheelchair each day (on average)?’ This was followed by: ‘From midday to when you go to bed, how many hours are you in the wheelchair each day (on average)?’ and ‘So overall in a day, you spend about (# hours) _____ in the wheelchair. Is that right?’ This last number was used for analysis. For analysis, responses were categorised as ‘Not daily’. ‘1–7 hours daily’ and ‘≥ 8 hours daily’.
Falls while in current chair	3-level (none, non-serious, serious)	Two survey items were ‘With your current wheelchair, have you ever fallen?’ and ‘Was this a serious fall? By serious, I mean a fall that left you with pain or soreness that lasted more than 1 h, bruising, skin cuts or abrasions, or injuries to your bones or joints?’ (q530 and q531) For analysis, one combined variable was coded to have three responses: ‘None’; ‘Falls, Non-serious’; and ‘Falls, Serious.’
Unassisted outdoor wheelchair use	Binary (yes, no)	Several survey questions were considered for this outcome (q402_a, b, and c). Users were coded as ‘yes’ on this outcome if they reached an area outside their home in a wheelchair, unassisted (code 0 on q401_c ‘did not need help’). Those who did not have another area to go to (possible answer for first question) were excluded (coded as missing). Those who reached another area but not in a wheelchair (i.e. on crutches) were coded ‘no’ (q401_a, b, and c).
Performance of activities of daily living (ADL)	Binary (high, low)	Four items measuring ADL: bathing or showering (q536a), dressing (q536c), eating (q536d), toilet hygiene (q536e) were used and summed. For analysis, this variable was split into high (3+) and low (0–2).
**Wheelchair services received**		
Assessment on 2+ aspects	Binary (yes, no)	The first step in creating this variable was determining whether the user was *asked* key questions by the provider: ‘Did the wheelchair provider measure or ask about your home environment (such as doorways and indoor spaces)?’ (q303_i) or ‘Did the wheelchair provider ask you about how and where you would use your wheelchair?’ (q303_j). The second step was asking about ‘expression’: ‘Did the wheelchair provider let you express your needs related to the wheelchair?’ (q303_c) or ‘Did the wheelchair provider listen to your needs and use the information you expressed?’ (q303_d). We added these two variables to the item, ‘Did the wheelchair provider measure your body?’ (q303_a). The score ranged from 0 to 3. The variable was then split into two levels: ‘Assessment on 2+ aspects’ was coded ‘yes’ for a score of 2 or 3 and ‘no’ for a score of 0 or 1.
Fitting (any)	Binary (yes, no)	This was a composite variable reflecting receipt of at least one of the following items with regard to the current or most recently acquired chair: Were you shown different types of wheelchairs or features to choose from? (q305) Did you have a choice from among a range of wheelchairs? (q308_a) Did you and your wheelchair provider agree on choice of wheelchair from the range of wheelchairs? (q308_b) Did you receive the wheelchair that you chose in agreement with the wheelchair provider? (q308_c) Did the wheelchair provider adjust or modify the wheelchair according to your needs? (q303_g)
Training	Binary (yes, no)	This was composite variable reflecting receipt of at least one of the following items: Did you ever receive any training related to the use of a wheelchair? (q312) During any training you have received, were the following addressed or not addressed? How to get around in a wheelchair (q315_a) How to get in and out of a wheelchair (q315_b) Preventing pressure sores, such as by performing pressure relief (leaning or lifting often) (q315_c)
Fit while propelling	Binary (yes, no) (1 item)	‘Did the wheelchair provider assess the fit of the wheelchair while you propelled the chair?’ (q303_e)
Provider asks or checks regarding skin	Binary (yes, no) (1 item)	‘Did the wheelchair provider ask you or physically check you for skin problems, sensation, or pressure sores?’ (q303_b)
Provider checks for unsafe pressure at seat	Binary (yes, no) (1 item)	‘Did the wheelchair provider check for unsafe pressure at your seat cushion surface (this would have required the assessor putting his/her hand under your buttocks)?’ (q303_h)
Assessment at home	Binary (yes, no) (1 item)	‘Did the wheelchair provider’s assessment or fitting occur at your home?’ q304
Duration of assessment	Binary (30+ min vs. < 30)	‘How long did the assessment take? This would include measuring your body, checking the fit of the wheelchair or making adjustments to the wheelchair.’ (q306)
Provider ever helped choose chair	Binary (yes, no) (1 item)	‘Has a wheelchair provider EVER helped you choose the right wheelchair? They might have measured your body, checked the fit of the wheelchair, or made adjustments to the wheelchair.’ (q309)
Instructions in maintenance	Binary (yes, no) (1 item)	‘Have you ever been instructed in taking care of your wheelchair, such as any of the following: keeping it clean, oiling moving parts, tightening spokes, and pumping tires?’ (q316)
Provider informed where to seek repairs	Binary (yes, no) (1 item)	‘Have you ever been told where to seek help with wheelchair repairs that you cannot manage yourself?’ (q319)
Provider followed up	Binary (yes, no) (1 item)	‘Has a wheelchair provider ever contacted you to ask how you are doing with a wheelchair since you received it?’ (q322)
Peer group training	Binary (yes, no) (1 item)	‘Have you ever received peer group training? This is a special training program me from other wheelchair users on several topics, usually not at the time that you received the wheelchair for the first time.’ (q521)
**Disability-related**		
Condition related to need for wheelchair	Several categories (dominant code selected)	‘What was the condition that led you to need a wheelchair?’ (q5) There were eight preformed response categories and ‘Other (specify).’ Many respondents gave an open-ended response that was later coded to these to the preformed or new categories. Some individuals gave more than one condition, and for these, the analysts determined which reason was dominant. In the Philippines, the categories were: spinal cord injury (paraplegic and quadriplegic), polio or post-polio, amputation, congenital disability, old age, arthritis, bone problems, stroke, nerve or clot, accident, infection, surgery or medical error or injection, muscle problems/weakness, and other. In Kenya, an additional category was diabetes. We report the major categories and collapsed for the inclusion in the multivariable analysis.
Where chair was obtained	Several categories	The survey item was ‘Where did you obtain your current wheelchair?’ (q105). Pre-coded response categories were: government unit (local or central or national); mission hospital; charitable organisation; church; pharmacy or medical supply store; given it by a friend or relative; and other (specify). The distribution of responses informed the categories used.
Type of wheelchair	Several categories	The data collectors were asked to record the type of wheelchair without asking the participants, as the data collectors had been trained to recognise the types. The preformed categories were: basic indoor chair and rough terrain chair (long wheel base). If the user was not in the current chair and the chair was unavailable, this was noted. ‘Don’t know’ was also possible.
Purchaser of chair	Several categories	‘Who paid for the chair?’ (q106) had eight response categories, and based on the distribution, this was reduced to ‘free of charge or no payment’ and ‘payment,’ so the variable became about whether payment was made.
Number of wheelchairs acquired in last 5 years	2-level (2+ vs. 0–1)	Number of wheelchairs acquired in last 5 years (q_10), a continuous variable, was generally responded to by most users with an answer of ‘1’. For analysis this variable was split at 2+ and 0–1. Some who responded ‘0’ were coded as missing because all respondents must have acquired a current chair in last 5 years to be included.
**Sociodemographic**		
Geography	Several categories	In Kenya, the counties were Kiambu, Machakos, Nairobi, Kajiado, Nakuru, Mombasa, Kisumu, Kisii, Eldoret and Kericho. In the Philippines, the local government units were Mandaluyong, Quezon City, Taguig, Las Pinas and Makati. An additional site of employment and residence of wheelchair users was used.
Age	Several categories	Age was noted by birth month and year (q1). Age did not appear to have a linear relationship with the outcomes and was believed to be more intuitive in categories, and was split at ages 18–34, 35–49 and ≥ 50.
Education	Several categories	‘What is the highest level of school you attended?’ (q3) Response categories were none/don’t know; primary; secondary, post-secondary, vocational, and college or university.
Marital status	Several categories	Marital status (q4) had categories of married, divorced or separated, widowed and never married and never lived together.
Work/ employment	Several categories	‘What kind of work do you mainly do now?’ (q509). Preformed response categories were: No work outside of home or unemployed (but not homemaker); homemaker or full-time parent; farming (agriculture, livestock); trading or selling; craftsman (e.g. carpentry, tailoring, masonry); office worker; student; labourer or casual worker; and other (specify). In Kenya, categories were collapsed to six upon review of the distribution of responses, while in the Philippines, as a vast majority of wheelchair users did not work, this was a dichotomous variable (did not work; work).
Wealth quintile	5 quintiles	Household wealth was based on many questions posed in large household surveys. Items having more than 5% of the sample (water source, toilet type, main type of fuel source, main floor type, main wall type, number of rooms and household assets such as electricity, radio, TV, mobile phone, refrigerator) were entered into a principal components analysis. The resulting variable was split into five equal groups or quintiles of wealth, representing a relative distribution of respondents from poorest to richest.

*Source*: Authors’ own work
