# Erratum: Wheelchair services and use outcomes: A cross-sectional survey in Kenya and the Philippines

**DOI:** 10.4102/ajod.v6i0.468

**Published:** 2017-12-13

**Authors:** Eva S. Bazant, Elizabeth J. Himelfarb Hurwitz, Brenda N. Onguti, Emma K. Williams, Jamie H. Noon, Cheryl A. Xavier, Ferdiliza D.S. Garcia, Anthony Gichangi, Mohammed Gabbow, Peter Musakhi, R. Lee Kirby

**Affiliations:** 1 Jhpiego, Baltimore, United States; 2 Jhpiego, Nairobi, Kenya; 3 Noon Design, Cerrillos, United States; 4 Private Sector, Matale, Sri Lanka; 5 College of Allied Medical Professions, University of the Philippines, Manila, Philippines; 6 National Council for Persons With Disabilities, Government of Kenya, Kenya; 7 Ministry of East African Community (EAC), Labour and Social Protection, Government of Kenya, Kenya; 8 Division of Physical Medicine & Rehabilitation, Dalhousie University, Canada

In the version of this article initially published, a sentence which appeared in the abstract needs to be corrected. The sentence reads ‘Conclusion: Select services that were associated with some better wheelchair use outcomes and should be emphasised in service delivery’ and should read ‘Conclusion: Select services were associated with some better wheelchair use outcomes and should be emphasised in service delivery.’ This correction does not alter the study’s findings of significance or overall interpretation of the study results. The publisher apologises for any inconvenience caused.

